# Integrating quantitative and qualitative approaches to assess wintertime illness-related absenteeism and its direct and indirect costs among the private sector in Ulaanbaatar

**DOI:** 10.1371/journal.pone.0263220

**Published:** 2022-02-03

**Authors:** Mandukhai Ganbat, Nasantogtokh Erdenebileg, Chuluunbileg Batbold, Saruultuya Nergui, Ron Anderson, Clarence Wigfall, Narantsetseg Amarsanaa, Alex Heikens, Moiltmaa Sarantuya, David Warburton

**Affiliations:** 1 Department of Epidemiology and Biostatistics, Mongolian National University of Medical Sciences, Ulaanbaatar, Mongolia; 2 School of Medicine, Mongolian National University of Medical Sciences, Ulaanbaatar, Mongolia; 3 Department of Applied Social Psychology, Claremont Graduate University, Claremont, California, United States of America; 4 Department of Accounting, Mandakh University, Ulaanbaatar, Mongolia; 5 United Nations Children Fund, Ulaanbaatar, Mongolia; 6 Department of Surgery, Children’s Hospital Los Angeles, University of Southern California, Los Angeles, California, United States of America; Universidad Pablo de Olavide, SPAIN

## Abstract

Causes for employee absenteeism vary. The commonest cause of work absenteeism is “illness-related.” Mongolia’s capital city, Ulaanbaatar, experiences high employee absenteeism during the winter than during other seasons due to the combination of extreme cold and extreme air pollution. We identified direct and indirect costs of absenteeism attributed to air pollution among private-sector employees in Ulaanbaatar. Using a purposive sampling design, we obtained questionnaire data for 1,330 employees working for private-sector companies spanning six economic sectors. We conducted 26 employee focus groups and 20 individual employer in-depth interviews. We used both quantitative and qualitative instruments to characterize the direct and indirect costs of absence due to illnesses attributed to severe air pollution during wintertime. Female employees and employees with a young child at home were more likely to be absent. Respiratory diseases accounted for the majority of reported air pollution-related illnesses. All participants perceived that air pollution adversely affected their health. Individual employee direct costs related to absence totaled 875,000 MNT ($307.10) for an average of three instances of three-day illness-related absences during the winter. This sum included diagnostic and doctor visit-related, medication costs and hospitalization costs. Non-healthcare-related direct cost (transportation) per absence was 50,000₮ ($17.60). Individual indirect costs included the value of lost wages for the typical 3-day absence, amounting to 120,000₮ ($42.10). These total costs to employees, therefore, may amount to as much as 10% of annual income. The majority of sick absences were unpaid. Overall, the cost of wintertime absences is substantial and fell disproportionately on female employees with young children.

## Introduction

Among the national capital cities, Mongolia’s capital, Ulaanbaatar, is the coldest on earth during the winter season. Many people survive winter by burning coal in domestic heating stoves, which produces >80% of the winter seasonal air pollution in Ulaanbaatar [1]. The extreme levels of PM_10_, PM_2.5_, SO2, and NO2 measured in the air are strongly associated with adverse health effects, including respiratory, cardiac and pregnancy-related morbidities [2–5]. Maji and colleagues found that long-term exposure to PM2.5 was connected to greater mortality in people aged >25 years from stroke, ischemic heart disease, and chronic obstructive pulmonary disease (COPD). At the same time, COPD caused 22.9% of PM2.5-attributable mortalities [6]. These detrimental effects are associated with substantial direct and indirect costs to employers, employees, and their children.

Absenteeism can be thought as a withdrawal behavior when people become physically and psychologically detached from the work [7]. In the United States (US) service sector, approximately 2.3% of entirely projected operative hours are missed due to unscheduled absences, mainly allotted to illness. Nevertheless, in other industries, the entire expense of unintentional absences might quantify as much as 20% of wages. Higher rate of non-attendance of working parents contributes as substantial capital cost [8].

In Oslo, Norway, 0.6% increment in employee sick-leave was linked with one unit rise in PM_10_ by 1μg/m^3^. Increased absenteeism entails, for the employee, a loss of sense of overall well-being and increased financial costs through more utilization of health services. The Norwegian results indicate that air pollution increased trade and industry costs through increased sick leave [9]. A study of indirect costs related to COPD showed that COPD is associated with very high indirect costs. Average annual indirect costs in US Dollars (currency symbol $) were $893–$2234/person with COPD. The disease also burdens employers because of lost productivity and associated costs and burdens individuals with lost income related to absenteeism, activity limitation, and disability [10]. A study of endometriosis costs in the US found that the additional direct and indirect twelve month costs per endometriosis patient were respectively $10,002 and $2132 [11].

Previous studies [12] have attempted to estimate the overall economic losses from exposure to air pollution in Mongolia. A World Bank report in 2011 estimated the health costs associated with Mongolia’s air pollution at $463 million (range $177 million–$727 million) per year, equivalent to 18.8% of Ulaanbaatar’s 2008 gross domestic product (GDP) [13]. The calculations were based upon a Value of Statistical Life of in Mongolian Tugrik (currency symbol ₮) of 319 million ₮ ($168,000), based on a local survey estimating Mongolians’ willingness to pay for mortality risk reduction in the winter of 2010. Similarly, a joint World Bank and Institute of Health Metrics and Evaluation report in 2016 concluded that approximately 2,424 Mongolians (adults and children) died prematurely in 2013 due to air pollution, totaling 4.1 trillion ₮ ($2.1 billion) in welfare losses (6.9% of Mongolia’s GDP). For that study, the country-specific Value of Statistical Life was calculated based on a base Value of Statistical Life of 7.5 billion ₮ ($3.83 million) for the Organization for Economic Co-operation and Development countries, adjusted by the per capita GDP with an income elasticity of 1.2 [12].

The United Nations Children fund report in 2016 on the Mongolian air pollution crisis established that air pollution-related diseases in children (ages 0–18 years) cost public health services in Ulaanbaatar 10.4 billion ₮ ($4.8 million) annually. When that analysis included adults, the costs increased to 18.4 billion ₮ ($8.5 million) per year [14]. Besides direct costs, missed schooling from air pollution-related diseases likely also adversely impacts Mongolian children’s education and future earnings. A worldwide analysis of the economic returns from education indicated that each additional year of schooling for Mongolian children is associated with a 9.1% average increase in future income [12]. The returns of completing primary, secondary and tertiary education averaged 13.4%, 4.2% and 10.1%, respectively, similar to nations in the East Asia region. There are also data that chronic school absenteeism (defined as missing more than 10% of school days in an academic year) increases the likelihood of dropping out of school, detrimentally impacting an individual’s future earnings [15].

The indirect costs of lost productivity in adults caring for sick children in Mongolia were also estimated. When the indirect costs associated with productivity losses of working parents, the total cost of air pollution-induced diseases increased to 19.6 billion ₮ ($9 million) in 2016 –equivalent to 2.9% of the Ministry of Health’s total budget that year and 8.7% of all health facility expenditures in Ulaanbaatar. Considering the value of working parents’ productivity losses, the total cost from 2017–2025 caring for 0–18-year-olds becomes 46.6 billion ₮ ($18.4 million)–approximately double the estimated direct costs. Annually, the total (direct plus indirect) cost of caring for 0–18-year-olds would be about 9 billion ₮ ($3 million) from 2021 onwards, which is over half of the total economic loss calculated for the entire population of Ulaanbaatar [14]. According to the report "Household Economic Research 2017", the average monthly expenses of a Mongolian household is 1 million ₮ ($354.30), of which 53,561₮ ($18.80) is spent on medical care [16].

Missing from the above summary are any Mongolian country-specific estimates of private employers and employees’ costs due to air pollution. We believe no such peer-reviewed information has been published. Consequently, we used mixed methods research combining qualitative and quantitative methods to assess their direct and indirect costs to employees and employers in Ulaanbaatar attributed to air pollution. We also studied the availability of flexible working arrangements during Mongolia’s highly polluted winter months.

## Materials and methods

### Study participants and sampling

Using a purposive sampling method, we defined target private-sector employers in Ulaanbaatar. In partnership with UNICEF, the study team identified target private-sector employees spanning the following 6 economic sectors: service, manufacturing, repair, financial, sales and professional. Inclusion criteria encompassed business type, employee numbers, and willingness to participate in the study. Companies were selected based on their number of employees and employer business type. Questionnaire data were obtained for 1,330 employees working for 9 private-sector companies. We conducted 26 focus groups and 20 individual employer interviews.

### Study design

This was a cross-sectional survey study type conducted between July 2018 to March 2019. In the study, we used mixed methods research, combining qualitative and quantitative research methods. In this study, the use of mixed methods contributed to determining direct and indirect costs related to employee and employer and the impact of flexible working arrangements.

### Data collection method

#### Questionnaire

Questionnaire data were obtained from 1,330 employees working for private-sector companies spanning six economic sectors. The questionnaire was developed as part of the study with input from three expert panels from public health, accounting and study methodology professors from Mongolia. Before the meeting with professors, we identified the content of items in the questionnaire and questionnaire format. While meeting with expert panels, we checked the validity of the terms readability, understandability and other concerns related to the questionnaire items. Lastly, fifty-two participants were included in a questionnaire pilot study before distribution to final participants. Socio-demographic information about the study participants, including wage data, was collected by questionnaire. The questionnaire was written and conducted in the Mongolian language. Absence information was assessed through five key questions: 1. Have you ever been absent due to illness experienced by you or your family members related to air pollution during wintertime? 2. How many days were you absent due to illness during air pollution? 3. How frequently were you absent due to illness during air pollution? 4. Who is the most vulnerable person in your family and may require your absence? 5. What type of absence do you incur due to illness during the winter air pollution period? There was no time limit to complete the questionnaire, and the average time to complete it was around fifteen minutes. It was clearly stated that the obtained information would remain confidential and would be used only for research. Our study participants were private-sector employees, and their employers approved the questions before distribution to the study participants. They were also informed that they could withdraw information before, during and after study. After questionnaire completion, the field researchers scanned the items and checked whether there were incomplete and double rated questions. The researcher kindly requested them to complete any unanswered questions before leaving. This minimized the number of missing values in the statistical analysis. The study questionnaire in both Mongolian and English language are attached as S1 File and dataset as S4 File.

#### Individual interviews

We assessed the flexible work arrangements related to absenteeism from the employee and employer perspectives. From the employer side, the study team used semi-structured, face-to-face interviews with human resource managers from among the selected companies to assess company compensation mechanisms associated with absenteeism. According to prior literature, the qualitative study sample size depends on the study’s goals, sample specificity, use of established theory, and quality of dialogue and analysis [17]. In this study, we extensively focused on the quality of each individual interview moderated by our senior expert. She guided all interviews, together with a separate note-taker who recorded the responses.

The employee side was assessed through a questionnaire and semi-structured individual face-to-face interview to understand flexible working conditions offered by employers and the stress related to getting absence approval from employers due to illness during wintertime air pollution. The questions in both Mongolian and English language are in S2 File.

#### Focus group interviews

There were a total of 133 people enrolled in 26 focus groups. Employee respondents in the focus group interviews were organized from 6 economic sectors with a maximum of 7 participants per group. Based on prior studies, the ideal size of a focus group for most noncommercial topics is three to six participants [18].

All interviews were audio-recorded in addition to the notes taken by the researcher. For further qualitative analysis, the interviews were transcribed in Mongolian and then translated into English. Textual analysis was performed by first identifying the keywords and sentences according to the questions, and then recurring ideas were grouped to construct codes, then themes. The investigators examined the category and sub-category heading titles, and short paragraphs were written summarizing findings for each sub-category, noting similarities and differences observed across groups. The questions in both Mongolian and English language are in S3 File.

### Direct and indirect cost estimation

Direct costs were estimated using the "cost of illness" approach developed by Jo et al. [19]. The focus of a cost of illness study is to determine and estimate all the costs of particular disease [20]. Cost of Illness (COI) studies provide a systematic way of communicating with the public and policy makers. Private employers (policy makers) and employees (public) both experience a variety of economic and medical harms associated with absenteeism [21]. Additionally, behavioral aspect of answering the questions of COI method is quite easy to obtain responses from study participants and the wording of questions is the most efficient to directly assess costs due to morbidity [22]. For fulfilling the goal, we used the cost of illness approach. There are two main categories of direct costs: medical costs and non-medical costs. Medical costs, also referred to as healthcare costs, include all healthcare costs directly related to the studied disease, from diagnosis and treatment to continuing care. In our study, direct medical costs of illness included hospitalizations, doctor visits, a health professional’s care, and prescriptions for any drugs. In contrast, transportation and food expenses related to the disease were estimated as non-healthcare-related costs. The direct costs were obtained from the self-assessment questionnaire. In the questionnaire, we asked the following questions: “Who is the sickest in your household during winter’s high pollution periods?” and for that person, “What is the frequency and average expense for diagnosis and doctor visits, purchasing medicine, hospitalization and food and transportation to obtain healthcare?” The median total direct costs per employee were then calculated as the sums of the above direct cost items as shown in Table 5.

Indirect individual cost appraisal [23]: To assess the indirect costs to the individual, a measure of productivity loss quantified the output lost due to cessation or reduction of the patient’s or family member’s productivity, as a consequence of morbidity, mortality, or disability caused by the disease under investigation. The study employed the human capital method, as it is one of the first formal methods developed to quantify life value. The hours of work lost by the person due to disease were estimated and multiplied by the hourly wage using this method. Hourly wage data was collected and multiplied by the number of hours or days the employee missed because of air pollution-related illness. For instance, if the company employee’s hourly wage was 5,000₮ ($1.76), and they missed work for three days (8 hours per day), the calculation was: 5,000₮ x (8 hrs x 3 days) = 120,000₮ ($42.10) for total lost wages. Each employee reported their salary within a range specified in the questionnaire.

### Ethics approval and consent to participate

Ethical approval was given by the Mongolian National University of Medical Sciences (#2019/03-13), and written and verbal informed consent was obtained from each participant. A list of the targeted organizations from the service, manufacturing, repair, financial, sales and professional sectors was complied. The United Nations Children’s Team contacted the appropriate managers of the targeted organizations in Ulaanbaatar and introduced our study’s nature and objectives. Each participant was assessed to verify they satisfied the inclusion criteria. We shared our study’s objectives with the participants and explained they could withdraw from the study at any time. We explained that the collected information would remain confidential and would only be used for scientific purposes.

### Statistical analysis

Summary statistics were reported as the mean ± standard deviation for variables with normal distribution and median (interquartile range) for skewed distribution variables. Categorical variables were presented as frequency and percentage. Pearson chi-square and Fisher’s tests were compared for absent-from-work and present-at-work groups’ demographic factors. The potential predictors of absenteeism studied, and how they were coded in the regression analyses are shown in Table 1.

**Table 1:** Coding of potential predictors of absenteeism for logistic regression.

Regression model variables	Variables type	Measurement	Reference value
**Dependent variables (Outcome)**			
Employee absenteeism	Categorical (Binary)	Absence	Presence
		Presence	
**Independent variables (Predictors)**			
Gender	Categorical (Binary)	Male	Male
		Female	
Age	Continuous	Years	
Having a child at home	Categorical (Binary)	Yes	No
		No	
Air pollution-related self-reported diseases	Categorical (Binary)	Yes	No
		No	
Company air pollution coping techniques	Categorical (Binary)	Yes	Yes
		No	
Body mass index (kg/m^2^)	Categorical (Binary)	Normal	Normal
		Above normal	
Do you use an air purifier at home?	Categorical (Binary)	Yes	Yes
		No	
If no, are you passive smokers at the workplace or home?	Categorical (Ordinal)	Never	Never
		Sometimes	
		Mostly	
Years worked for current employer	Categorical (Binary)	< 5 years	< 5 Years
		≥ 5 years

$${Log}_{e}\left(odds[absenteeism]\right)=\beta_{0}+\;\beta_{i}X_{i}+\varepsilon_{i}$$ (a)

Where,

log_e_—Dependent variable (Employee absenteeism),

β_0_—Intercept: Log odds of employee absenteeism when all independent variables are zero.

β_i_—Odds ratio assocciated with each independent variable (Table 1)

ε_i_—Random error component for each independent variable

$${Log}_{e}\left(odds[absenteeism]\right)=\beta_{0}+\beta_{1}X_{1}+\;\beta_{i}X_{i}+\varepsilon_{i}$$ (b)

Where,

log_e_—Dependent variable (Employee absenteeism),

β_0_—Intercept: Log odds of employee absenteeism when all independent variables equal zero

β_1_—Odds ratio assocciated with female

β_i_—Odds ratio assocciated with all other independent variables’ values (Table 1)

ε_i_—Random error component

We first used univariate logistic regression to test the association between absenteeism and demographic factors potentially associated with absenteeism. The coefficient from each univariate result was reported as a crude odds ratio with its respective 95% confidence intervals (Eq a). A multivariate logistic regression analysis was then done using the backward elimination technique to account for the relationship between the independent predictors and potential confounders (Eq b). We started the analysis with the statistically significant independent variables identified by univariate logistic regression and used a standard elimination threshold of p ≥ 0.01. The coefficients from the multivariate results were reported as adjusted odds ratios with their respective 95% confidence intervals. All independent variables satisfied the independence of observations assumption of logistic regression, and there were no significant outliers.

Missing data comprised less than 5% for each variable, and list wise deletion was applied when during the analyses. Statistical significance was set at p < 0.05, and 2-tailed tests were used. The analyses were performed using IBM SPSS 26.0.

## Results

### Quantitative results

Employee demographic characteristics are shown in Table 2. Employees were distributed across six private business sectors. Their Median age was 31 years, and half were female. Their domiciles were distributed across 4 Khoroos (administrative districts) in Ulaanbaatar. Nearly 80% of employees had received higher education. The median salary was 500,000₮ ($175.50) per month, while 80% of employees had two children at home. (Mongolian Tugrik currency exchange rate for US Dollar on January 1, 2019, was 2644₮ /$1).

**Table 2:** Socio-demographic characteristics of study participants (n = 1330).

Variables	N	%
**Study employee number by service type (n = 1330, 100%)**		
Service sector employee	248	18.7
Manufacturing sector employee	257	19.3
Repair sector employee	182	13.7
Financial sector employee	521	39.2
Sales sector employee	61	4.6
Professional sector employee	61	4.5
**Gender (n = 1330, 100%)**		
Male	665	50.0
Female	665	50.0
**Age** (years, mean ± sd), **(n = 1330, 100%)**	31.0 ± 8.0	
**District where they live (n = 1330, 100%)**		
Bayanzurkh	368	27.4
Bayangol	258	19.4
Songinokhairkhan	344	25.9
Khan uul	175	13.2
Others	185	14.1
**Education status (n = 1330, 100%)**		
University graduate	1013	78.8
High school graduate	267	20.8
Did not graduate	50	0.4
**Children (n = 1330, 100%)**		
Yes	1075	80.8
No	255	19.2
**Number of children** (mean ± sd), **(n = 1330, 100%)**	2 ± 1	

^a^ Study participant’s data collected during the study and consistent with National Statistical Office data 1212.mn. ₮—currency symbol for Mongolian Tugrik; $—currency symbol for United States Dollar

**Fig 1:**
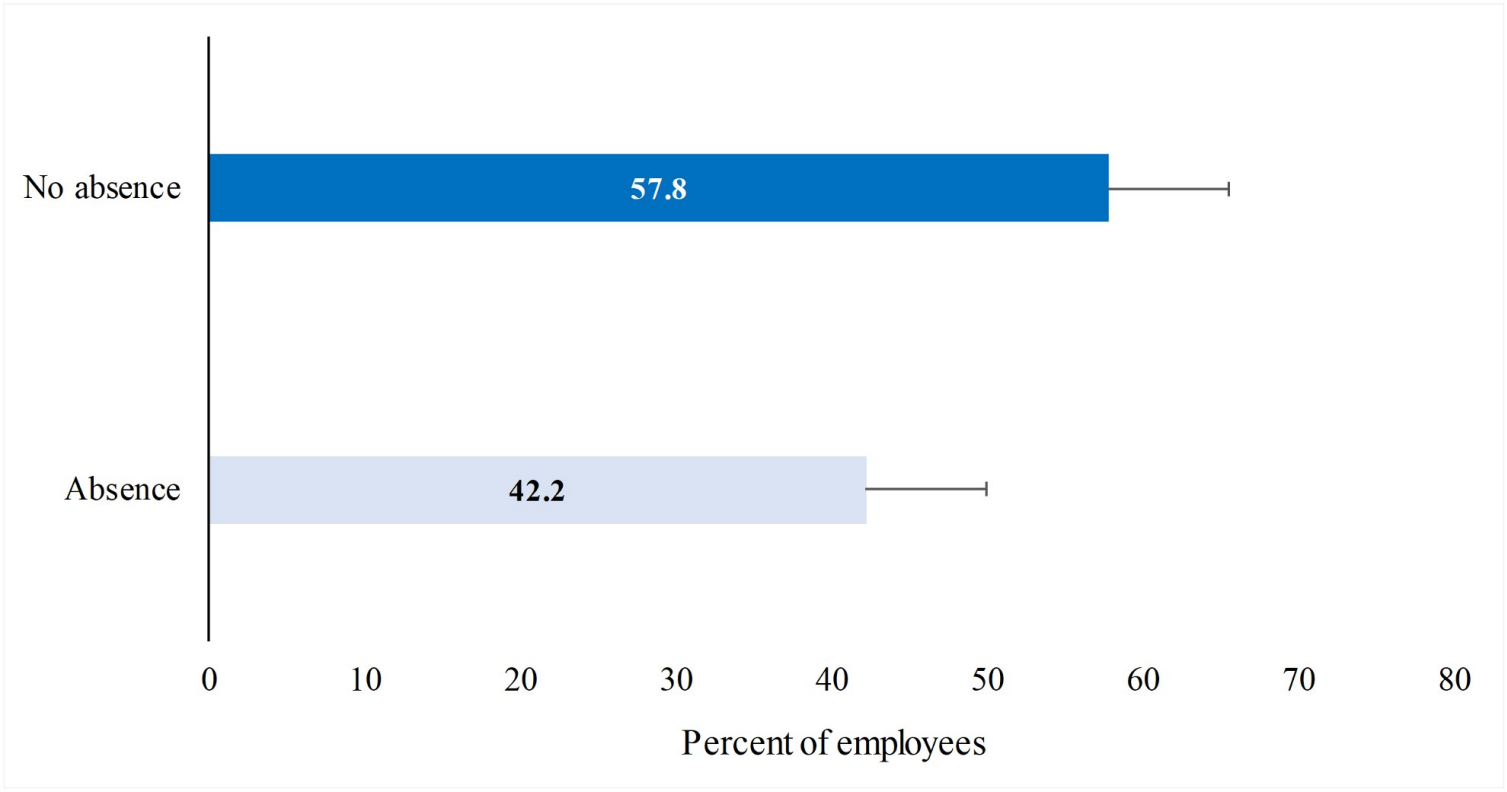
Self-reported wintertime absenteeism rate attributed to wintertime air pollution among study selected participants (n = 1330).

Fig 1 shows the proportion of employees who took at least one absence during the winter season 2018–2019 (58%) based on the survey results.

**Fig 2:**
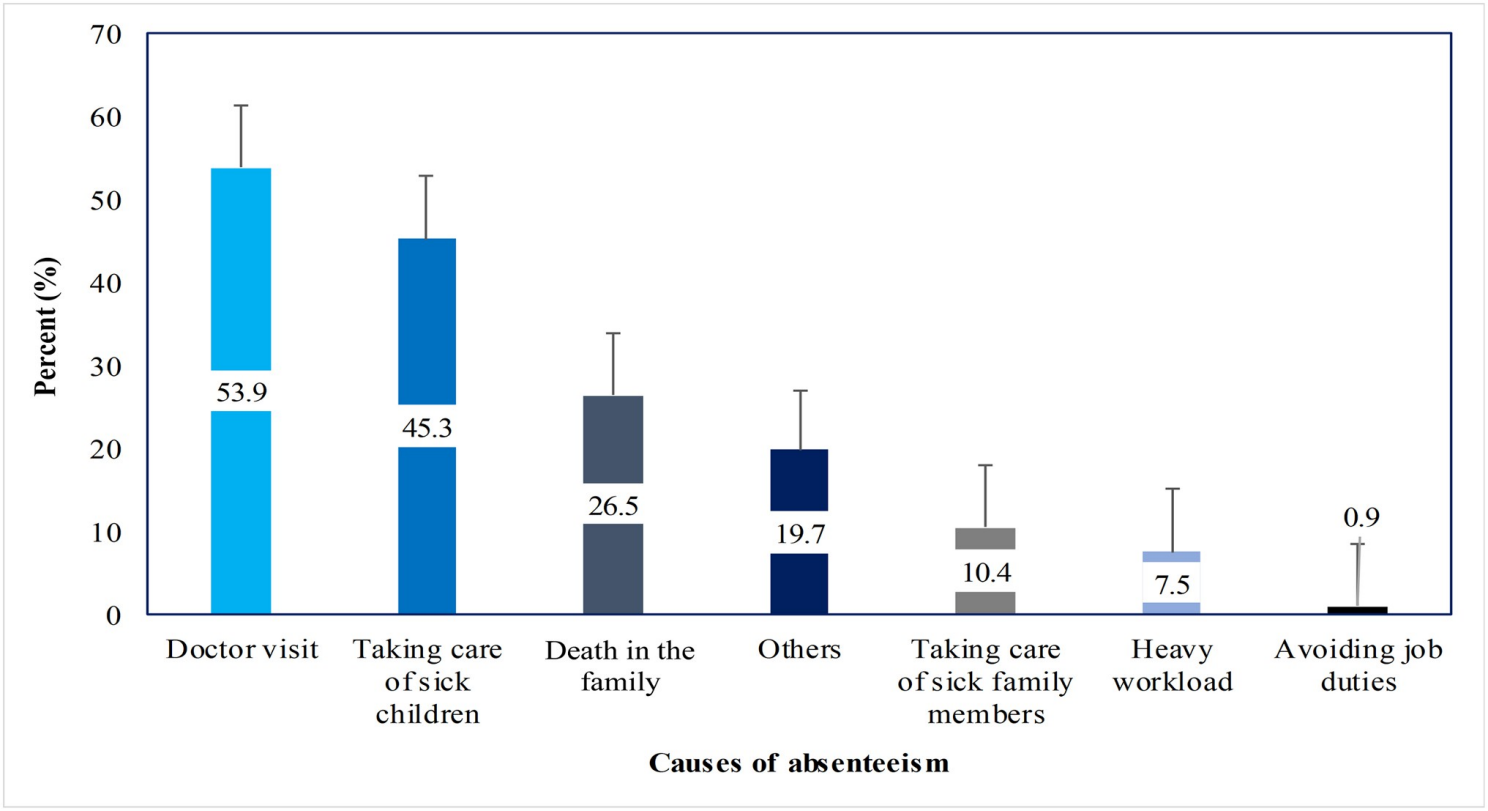
Causes of absenteeism attributed to wintertime air pollution.

Fig 2 shows employees’ main reasons to justify absences from work, including doctor visits because of being sick (54%) and taking care of sick children (45%). Importantly, death in the family was also given as a justification by 27%.

Table 3 quantifies company policies and employee perceptions of company flexible care policies for sickness-related absences. Almost forty percent of the private company employees who participated in this study had experience coordinating (covering) co-workers’ duties when they are out sick during the winter air pollution period. The same percentage of employees reported being stressed due to the extra work when others are unexpectedly absent. Eighty percent of study participants in the private sector reported mixed feelings of frustration and fear when asking for sick leave during winter. Seventy-five percent of the participants responded that there were few or no flexible time arrangements from their employer.

**Table 3:** Employee perceptions regarding the impact of sickness-related absences and the availability of flexible working arrangements from questionnaire data from 1,330 employees working for private-sector companies spanning six economic sectors.

QUESTIONS	N	%
**How often do you coordinate the duty of co-workers when they are sick during the high air pollution period? (n = 1072)**		
Mostly	144	13.4
Sometimes	276	25.7
Rarely	333	31.1
Never	319	29.8
Total	1072	100.0
Missing value	258	19.3
**If yes, have you ever been stressed due to assuming an unplanned missing person’s role at your job?**		
Mostly	156	13.9
Sometimes	261	23.2
Rarely	296	26.3
Never	412	36.6
Total	1125	100.0
Missing value	205	15.4
**Feeling when you request sudden leave from your job due to sickness during high air pollution (single response)**		
Worried	299	26.0
Scared	599	52.1
Relaxed	193	16.8
Other	58	5.1
Total	1149	100.0
Missing value	181	13.6
**Does your company have flexible working arrangements such as working from home or duty sharing?**		
Mostly	113	11.0
Sometimes	143	13.9
Rarely	146	14.2
Never	625	60.9
Total	1027	100.0
Missing value	303	22.7

**Fig 3:**
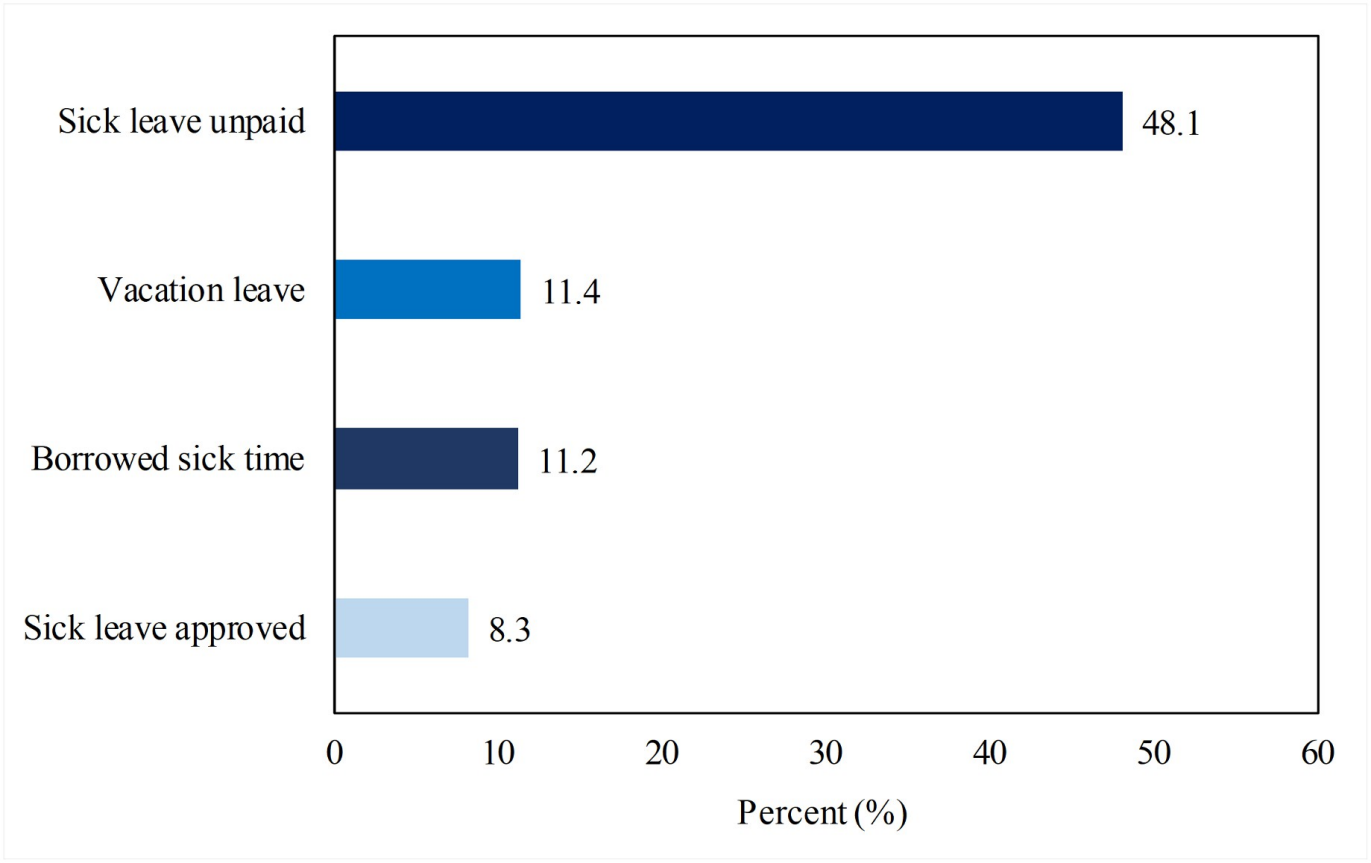
Types of temporary leave among study participants.

Fig 3 shows that sick leave was approved for about a third of the employees who asked for it. Nearly half of employees did not receive sick pay for absences, while a small proportion had an option to use sick day leave or vacation leave as offered by their company.

Table 4 shows that female gender and having children are significant risk factors for workplace absences during the winter pollution season. The predictor variables were tested to verify the multicollinearity assumption was tenable and a correlation matrix generated. The deviance goodness-of-fit test indicated that the model was a good fit to the observed data, χ2 = 5849.31, p = 0.024 in crude and χ2 = 9.6, p = 0.032 in adjusted analysis. A female was 1.65 times more likely to be absent than her male counterparts. Even after adjustment for other variables, female gender remains a significant factor for air pollution-related workplace absence (p = 0.03; 95 CI 1.04–2.54). Reporting having a child imparts 1.87 times higher odds of being absent than persons without children adjusted by other factors (p < 0.001; 95 CI 1.87–4.49). Both self-reported diseases and company air pollution coping techniques were 1.17 and 1.64 times more associated with absence. However, these risk factors became non-significant after adjusting for confounders (p = 0.08; 95 CI 0.87–10.97 and p = 0.19; 95 CI 0.91–2.36). Being overweight and obese were not associated with being absent compared to the normal BMI group. Lastly, being a passive smoker and working for a company greater than five years were significant factors associated with absenteeism.

**Table 4:** Potential absenteeism risk factors from questionnaire data from 1,330 employees working for private-sector companies spanning six economic sectors.

Variables	Crude OR^a^				Adjusted OR^b^			
	OR	95%, CI		P-value	OR	95%, CI		P-value
		Lower	Upper			Lower	Upper	
**Gender**								
Male	1				1			
Female	1.65	1.29	2.11	0.001	1.63	1.04	2.54	.**03**
**Age** (p-value)	0.002 per year (0.319)							
**Having a child at home**								
Yes	2.23	1.62	3.07	0.001	2.90	1.87	4.49	.**001**
No	1				1			
**Air pollution-related self-reported diseases**								
Yes	1.17	0.90	1.52	0.23	3.08	0.87	10.97	.08
No	1				1			
**Does your company use air pollution coping techniques?**								
Yes	1				1			
No	1.64	1.10	2.44	0.01	1.46	0.91	2.36	.19
**Body mass index**								
Normal (18.5 to <25)	1							
Above normal (≥25)	1.10	0.94	1.45	0.09	1.12	0.98	1.65	.12
**Do you use an air purifier at home?**								
Yes	1				1			
No	1.11	0.82	3.4		1.28	0.79	4.03	.16
**If no, are you passive smokers at the workplace or home?**								
Never	1				1			
Sometimes	1.18	0.64	2.12		1.45	0.74	2.84	.279
Mostly	1.15	0.74	1.78		1.21	0.74	1.93	.41
**Years worked for current employer**								
< 5 years	1				1			.**01**
≥ 5 years	1.36	1.05	1.75		1.39	1.11	1.62	

^a^Crude odds ratio from the univariate logistic regression coefficient testing the association between absenteeism and the factor.

^b^Adjusted odds ratios from the multivariate regression coefficients testing the association between absenteeism and the significant univariate factors using the backward elimination technique with p ≥ 0.10 as the elimination threshold.

Table 5 shows the contributing components to employees’ direct costs for medical-related absences during the wintertime air pollution season. All participants believed that air pollution adversely affected their health. Individual employee direct costs related to absence totaled 875,000₮ ($307.10) for an average of three instances of three-day illness-related absences during the winter in Ulaanbaatar. This sum included diagnostic and doctor visit-related costs per absence of 65,000₮ ($22.80) (three times), medication costs per absence of 70,000₮ ($24.60) (four times), and hospitalization costs per absence of 200,000₮ ($70.20). Non-healthcare-related direct cost (transportation) per absence was 50,000₮. Individual indirect cost equated to the median value of lost wages for a 3-day absence amounting to 120,000₮. Thus, the costs to employees may amount to as much as 10% of their annual income.

**Table 5:** Individual healthcare and non-healthcare-related direct costs per employee per winter season attributed to air-pollution-related illness. Data are from a survey of 1,330 employees working for private-sector companies spanning six economic sectors.

Categories of healthcare-related direct costs	Median frequency of healthcare cost events per employee	Median cost per healthcare event	Total median cost of healthcare events per employee
**Individual healthcare-related direct costs** ^a^			
Diagnostic services and doctor visit-related costs	3	65,000₮ ($22.80)	195,000₮ ($68.40)
Medication purchasing-related costs	4	70,000₮ ($24.60)	280,000₮ ($98.30)
Hospitalization-related costs	1	200,000₮ ($70.20)	200,000₮ ($70.20)
**Individual non-healthcare-related direct costs** ^b^			
Transportation	4	50,000₮ ($17.60)	200,000₮ ($70.20)
Total employee direct costs			875,000 ₮ ($307.10)

^a^Direct medical costs of illness incurred per employee.

^b^Transportation costs incurred by the employee.

The total cost was estimated by multiplying the event frequency by the event cost. ₮—currency symbol for Mongolian Tugrik; $—currency symbol for United States Dollar

Table 6 described the effect of absenteeism on human capital costs. This calculation revealed the substantial increases in human capital costs driven by increases in days of absence. The median indirect cost due to three missed days of work was 120,000₮ ($42.10) in this study (95% CI 80,000–210,000₮) ($28.10–$73.70). One missed day cost 35,000₮ ($12.30).

**Table 6:** Individual indirect costs attributed to air pollution-related illness per employee per winter season using the human capital approach. Data are from a survey of 1,330 employees working for private-sector companies spanning six economic sectors.

		95.0%, CI		Interquartile	
Variables	Median	Lower	Upper	25^th^	75th
Number of days absent	3	3	5	2	7
Lost salary due to one day missed	35,000₮ ($12.30)	30,000₮ ($10.50)	40,000₮ ($14.10)	25,000₮ ($8.80)	50,000₮ ($17.60)
Individual indirect cost due to absenteeism^a^	120,000₮ ($42.10)	80,000₮ ($28.10)	210,000₮ ($73.70)	60,000₮ ($21.10)	245,000₮ ($85.90)

^a^Estimated days of work lost by the employee due multiplied by the daily wage;

CI—Confidence interval; ₮—currency symbol for Mongolian Tugrik; $—currency symbol for United States Dollar

### Qualitative results

Qualitative research through individual interviews was used to provide better insight into individual and company absenteeism costs during Ulaanbaatar’s wintertime air pollution-related sickness.

#### Some factors related to absence from the work

From the employees’ perspective, child sickness was the most common reason given to take a day of leave. Child Illness occurred at least one to three times in the winter season, and the frequency of sickness increased in wintertime. Employees believed there were various reasons for child’s vulnerability to air pollution such as; child age, child sensibility to the toxic effect of pollution, overcrowding of classroom (at kindergarten and or school) and the speed of infection spread. One employee said: “*The air below 1 meter*, *which is the same height as most children*, *is more polluted*, *so as the children get sick more often*,*”* and another said, *“The frequency of child sickness has a direct relation with the number of children in a family; if one is sick the other most probably get sick too*.”

Female workers take more time for sick leave due to their children’s sickness than male workers. One male employee said, “When our child gets sick, I leave my sick child and my wife at the hospital, and my wife stays there, so I go back to work.”

Conversely, if employees find themselves sick, they continue attending their jobs so they would not miss their hourly wage. In rare cases, they take several hours off or the entire day off to visit a doctor.

Almost all workers subtract their absent days from their residual vacation days, or they try to schedule their vacation in wintertime when child sickness is frequent. If they cannot subtract their sick leave from their vacation, they take an unpaid absence unless they can bring a sick note from a physician. In 2017, Resolution No. 215 Mongolian Government granted paid sick leave for parents who need to take care of their sick children under age 5. Employees typically ask someone, often their parents, or hire somebody to taken care of their child.

#### Cost of absence

The payment is paid from health insurance for employees who receive pediatrician consultation from a public healthcare facility. However, the majority receive a consult from private clinicians with out-of-pocket payment due to being unsatisfied with public health facilities regarding doctor’s experience and skills, accessibility and lack of advanced technology. One of the employees commented that “*Physicians in public family doctor clinic are usually recently graduated young girls who have not much work experience*, *so I do not trust them*. *Moreover*, *the treatment I received was not effective enough*, *so I had to consult with another doctor later*”.

On the other hand, some private health centers make appointments on weekends and offer second consultation free of charge within 14 days. However, there are other facilities where 20,000₮ ($7.00) is charged for a follow-up consultation. The cost for laboratory tests starts from 50,000₮ ($17.51) and reaches a maximum of 200,000₮ ($71.90). For radiology tests, the price varies between 15,000₮ ($5.25) to 300,000₮ ($105.09), and some health providers in public health facilities request X-ray imaging, which must be done at private facilities. When the child becomes ill, employees seek faster resolution of sickness, so they purchase oral or injectable antibiotics over-the-counter at a pharmacy, without medical advice. For injections, parents pay an on-call nurse to do it with a payment of 5,000₮ ($1.75) added for each daytime call. Employees also mentioned that they change the medicine on the third or seventh day after initiating the treatment if they do not see any improvement. This leads them to buy another drug and waste the previous one. The practice of treating cough with vitamin C, seabuckthorn or lingonberry juices, and fermented hot, dry milk at home was prevalent among employees.

From employer perspectives, the absence of one employee can bring various opportunity costs, including delayed service or product delivery due to the workforce deficit. For instance, only a few individuals reported that their company could not find substitutes in the absence of employees in the short term. In contrast, others resolve this problem by transferring absent an employee’s work to another employee or postpone the execution of their duties.

Almost all companies incur a substitute’s training cost if a current employee’s absence is more than one month. During wintertime, employees overtime costs increase, one employer said. Company production loss is measured by different methods depending on the function of the particular business, such as client complaints, the penalty for delay of product, postponing scheduled appointment or delay of the project, or sometimes this is calculated by score and results in reduced salary. Company leaders also remarked that worker’s productivity declines after 8 hours of work, and overload of work also reduces productivity.

## Discussion

The main findings of our study relate to employee absenteeism due to reported genuine illness and its costs. Illness-related work absence is costly to employers who offer sick leave because employees are paid for time when they are not working. It is likewise costly to employees without sick leave due to lost wages. Regardless of sick leave coverage, absenteeism is always associated with work not being accomplished, delayed, or shifted to others and is associated with increased healthcare expenses.

Sick leave is a benefit provided to employees, which allows them to recover from illness [24]. Yet its usage often spills over to absenteeism related to care for a sick family member. When a child is sick, childcare costs can become a significant concern, as confirmed by our data. Another challenge common among families is the obligation of adults to care for their aging parents. These obligations often include staying home when a parent is ill, trips to the doctor, lab and imaging tests, hospitalizations, etc., which all require time away from work to accomplish [25].

Many employers use health advocacy schemes to restrain growing employee-related healthcare expenses, primarily because of direct medical and indirect costs [26]. In 1999, the growth of healthcare expenses for US companies averaged 8.3%, with nearly all of the growths paid by employers. In this identical year, company-paid yearly healthcare expenses increased for single-person insurance to $2426 and for family insurance to $6351. As companies face these rising costs, they continue to seek further ways to decrease employee-related healthcare cost. Whether increased work hours results in increased stress leading to more absenteeism persists unreadable [27].

In previous studies estimating costs of air pollution, authors used standard survey estimating the willingness to pay for reduction of health risks associated with air pollution. They converted willingness to pay into a value of statistical life estimate. The value of statistical value is the average willingness to pay for a small-scale abatement in individual mortality risk states in respect of risk to a population and represents the sum of individuals’ willingness to pay for marginal reductions in their mortality risks. The willingness to pay based approach is convenient for economic welfare analysis and valuing mortality risks related to pollution, especially in high income countries [12, 13]. We used COI approach measuring medical and other costs resulting from specific diseases related to air pollution. The COI approach is convenient for the study frame and economic analysis for estimating healthcare related direct costs.

Our research showed strikingly higher absenteeism by female employees with young children. The number of children in the family also had a significant impact on workplace absenteeism in our study. In Mongolia, women generally take more responsibility for child-rearing and are therefore more frequently absent, particularly as the number of young children in the family grows [28].

In our study, we were unable to collect the employees’ medical claims. However, Tomohisa et al. in Japan listed thirty-four health conditions and their annual estimated cost due to presenteeism, the practice of coming to work despite illness, anxiety, injury, etc., frequently resulting in lost productivity. Their study identified the following as primary sources, per person, with diagnosis, ICD10 code, and cost: insufficient sleep (F00F99) $341.58, anxiety (F41.9) $230.45, and cold, influenza (J00J99)$ 35.06 [29].

The studies of Loeppke and others have done in the US determined the top 10 health conditions by cost, including absenteeism and presenteeism, medical and pharmaceutical expenses. Mental health conditions were a substantial proportion of presenteeism costs. Partial of the absenteeism costs were due to the medical expenses, although in Japan, wholly cost was attributed to presenteeism [30].

Other studies, like that of Anderson et al., utilizing the HERO database, identified that nearly 8% of the total healthcare spending for one community of 46,000 people was independently correlated with decreased emotional well-being [31]. Manning et al. similarly found that stress and insufficient livelihood welfare accounted for 7% to 9% of entire medical costs [32].

Our study’s weaknesses include the possibility of recall bias in focus group interviews, socioeconomic selection bias because the subjects were all employed and better-educated and thus may under or overestimate the potential out-of-pocket GDP impact on employees in the Mongolian population. The representativeness of the study was limited by the purposive sampling method. We studied the direct and indirect costs associated with absenteeism, which our study participants attributed to air pollution. We did not identify the extent to which confounding variables, such as wintertime respiratory infections, affected these costs.

## Conclusions

We conclude that the identified combined direct and indirect costs of absenteeism attributed to wintertime air pollution in Ulaanbaatar, Mongolia, is compelling and substantial. The primary drivers of direct costs to employees are healthcare expenses, including doctor visits, medication, hospitalization, transportation, and the cost of loss of earnings. Although employees were equally divided by gender, the cost of illness-induced absences falls disproportionately on female workers with young children. The major cost drivers to employers are the number and human capital cost of employee absences.

## Supporting information

S1 File The study questionnaire in both Mongolian and English language. (DOCX)

S2 File The study individual interview questions. (DOCX)

S3 File The study focus group interview questions. (DOCX)

S4 File The study dataset. (XLSX)
