# Supplementary material for: Integrating quantitative and qualitative approaches to assess wintertime illness-related absenteeism and its direct and indirect costs among the private sector in Ulaanbaatar
Source: PLoS One. 2022 Feb 3;17(2):e0263220. doi: 10.1371/journal.pone.0263220 (PMC8812901; doi:10.1371/journal.pone.0263220)
Supplement: S1 File — (DOCX) [file pone.0263220.s001.docx]

# Questionnaire in english

ID _________

| **№** | **Opening Questions** | | **Answer Section** | | | | Code |
| --- | --- | --- | --- | --- | --- | --- | --- |
| **Opening questions** | | | | | | | |
| 1 | Were you born in Ulaanbaatar? | | 1. Yes 2. No | | | | К1 |
| 2 | How many years have you been living in Ulaanbaatar?  **(Please do not count years living in other places)** | | ______ year/years | | | | К2 |
| 3 | Do you think that air pollution causes diseases? | | 1. Yes 2. No | | | | К3 |
| 4 | In which season of the year do you get the sickest? | | 1. Winter 2. Spring 3. Autumn 4. Summer | | | | К4 |
| **Common symptoms related to air pollution** | | | | | | | |
| 1 | Which symptoms have you experienced during high air pollution periods?  **(Multiple answers)** | | | | 1. Cough 2. Chest pain 3. Sore throat 4. Scratchy throat 5. Itchy nose 6. Blocked nose 7. Runny nose 8. Shortness of breath 9. Difficulty breathing 10. Blurry vision 11. Sore eyes 12. Red eyes 13. Itchy eyes 14. Runny eyes 15. Dizziness 16. Headache 17. Heavy head 18. Fatigue 19. Nausea | | Q1 |
| 2 | Which diseases have you and your family experienced during high air pollution periods?  **(Multiple answers)** | | | | 1. Cough 2. Allergy 3. Bronchitis 4. Pneumonia 5. Heart attack 6. Hypertension 7. Diabetes 8. Chest pain 9. Congenital abnormality 10. Miscarriage 11. Premature infant death 12. Low birth weight 13. Spontaneous abortion 14. Stroke 15. Cerebrovascular disease 16. Migraine | | Q2 |
| **Air pollution exposure and costs** | | | | | | | |
| 3 | Have you and your family ever been absent during high air pollution periods? | | | | 1. Sick child 2. Sick parent 3. I was sick 4. Not absent even when sick 5. None | | Q3 |
| 4 | If yes, what was the disease? | | | | ___________________________ | | Q4 |
| 5 | How long have you been sick due to air pollution-related diseases on high pollution days? | | | | 1. In hours__________________ 2. In days________________ 3. Still come to work even when sick 4. I have never been sick | | Q5 |
| 6 | How many times are you absent from work during the high air pollution winter season? | | | | 1. None 2. 1 time 3. 2 times 4. 3 times 5. 4 times or more | | Q6 |
| 6.1 | How much is your salary deducted per day when you miss work? | | | | ___________ | | Q6_1 |
| 7 | What kind of temporary leave do you request when you are sick during high air pollution levels?  **(Multiple answers)** | | | | 1. Sick leave (paid) 2. Sick leave (unpaid) 3. Borrowed sick time 4. Vacation leave | | Q7 |
| 8 | How often are you responsible for the duties when they are sick during high air pollution periods? | | | | 1. Mostly 2. Sometimes 3. Rarely 4. Never | | Q8 |
| 9 | If yes, have you ever felt stressed due to unplanned roles at your job? | | | | 1. Mostly 2. Sometimes 3. Rarely 4. Never | | Q9 |
| 10 | How do you feel when you request sudden leave from your job due to illness during high air pollution in winter? | | | | 1. Worried 2. Scared 3. Relaxed 4. Other | | Q10 |
| 11 | Does your company have flexible working arrangements, such as working from home or duty sharing? | | | | 1. Most of the time 2. Sometimes 3. Rarely 4. Never | | Q11 |
| 12 | Who is the sickest in your household during winter's high air pollution periods? | | | | 1. Myself 2. Pregnant wife 3. Spouse 4. Kids 5. Parents 6. Grandparents | | Q12 |
| For that person, what are the frequency and average expense for each item below? | | | | | Answer | |  |
|  |  |  |  |  | Frequency (times) | Average costs (MNT) |  |
| 13 | | Diagnosis and doctor visits | | | _ _ _ | ___ ___ ___ ___ | Q13A |
|  |  | Purchasing medicine | | | _ _ _ | ___ ___ ___ ___ | Q13B |
|  |  | Hospitalization | | | _ _ _ | ___ ___ ___ ___ | Q13C |
|  |  | Transportation and food | | | _ _ _ | ___ ___ ___ ___ | Q13D |
| **Demographic information** | | | | | | | |
| 14 | | Age | | _______________ | | | Q14 |
| 15 | | Gender | | 1. Male 2. Female | | | Q15 |
| 16 | | Education | | 1. University 2. High school 3. Elementary school | | | Q16 |
| 16_1 | | Years of education | | ___________(how many years) | | | Q16_1 |
| 17 | | Current position | | _______________ | | | Q17 |
| 17_1 | | What is your monthly salary range? | | 1. <399,000 2. 400,000- 699,000 MNT 3. 700,000- 899,000 MNT 4. 1,000,000 – 1,299,000 MNT 5. 1,300,000 – 1,599,000 MNT 6. 1,600,000- 1,899,000 MNT 7. 1,900,000 - 2,199,000 MNT 8. >2,200,000 MNT | | | Q17A |
| 18 | | Years and months working for your current employer | | ________ years / _____ months | | | Q18 |
| 19 | | Home address  (Home address will be used to assess individual exposure level) | | ____________________ District   ____________khoroolol_______ khoroo_____Apartment/ House_______ Street_______Door Number | | | Q19 |
| 20 | | Do you have any children? | | 1. Yes  2. No  Fem | | | Q20 |
| 21 | | If yes, how many children do you have? | | _______ | | | Q21 |
| 22 | | How many hours do you work per day? | | ______ | | | Q22 |
| 23 | | Distance from home to office | | ______ kilometres  Number of bus stops: ___ | | | Q23 |
| 24 | | How often do you wear an air pollution mask during the high air pollution winter season? | | 1. Mostly 2. Sometimes 3. I wear it when I remember 4. Never | | | Q24 |
| 25 | | Do you use an air purifier at home during the high air pollution winter season? | | 1. Mostly 2. Sometimes 3. Rarely 4. Never | | | Q25 |
| 26 | | Please choose that what actions your company takes to protect employees from air pollution’s harmful effects? | | 1. Has never taken action 2. Distributed air pollution masks 3. Distributed air purifiers for each room 4. Distributed hot dried curds, sea buckthorn juice 5. Don’t know 6. Other___________________________ | | | Q26 |
| 27 | | Which type of residence do you live in? | | 1. *Ger* 2. *Ger* district house 3. Apartment 4. Other_____ | | | Q27 |
| 27_1 | | What kind of heating and fuel sources do you burn?  (Only applicable for those living in a *ger* or *ger* district house) | | 1. Processed coal 2. Raw coal 3. Wood 4. Cow dung 5. Other | | | Q27_1 |
| 28 | | What kind of transportation do you use when you go to work? | | 1. By walk 2. By bicycle 3. By car 4. By bus 5. By organization’s transportation for employees 6. Others | | | Q28 |
| 29 | | Do you smoke? | | 1. Yes 2. No | | | Q29 |
| 29_1 | | If yes, how many years and how many cigarettes per day? | | By yea Years ______ Number of daily cigarettes______ | | | Q29_1 |
| 29_2 | | If no, are you a passive smoker at your workplace or at home? | | 1. Usually 2. Sometimes 3. Never | | | Q29_2 |
| 30 | | Your body weight and height?  (weight and height will be used to calculate body mass index) | | 1. Height______cm 2. Weight_______kg | | | Q30 |

# Questionnaire in MONGOLIAN

ID _________

| **№** | **Эхлэх асуултууд** | | **Хариулт сонгох хэсэг** | | **Код** |
| --- | --- | --- | --- | --- | --- |
| **Эхлэх асуултууд** | | | | |  |
| 1 | Та Улаанбаатар хотод төрсөн үү? | | 1. Тийм 2. Үгүй | | К1 |
| 2 | Улаанбаатар хотод нийт **хэдэн жил** амьдарч байна вэ?  **(Өөр газар амьдарсан жилүүдийг оруулахгүй.)** | | ___ ___жил | | К2 |
| 3 | Та агаарын бохирдолтой холбоотойгоор өвчлөл ихэсдэг гэдэгтэй санал нийлдэг үү? | | 1. Тийм 2. Үгүй | | К3 |
| 4 | Та **аль улиралд** хамгийн өвчлөмтгий байдаг вэ? | | 1. Өвөл 2. Хавар 3. Намар 4. Зун | | К4 |
| **Агаарын бохирдлын үед илэрдэг нийтлэг шинжүүд** | | | | | |
| 1 | Танд агаарын бохирдол ихтэй үед илэрдэг **шинжүүдийг** сонгоно уу.  **(Та олон хариулт сонгож болно.)** | | 1. Ханиалгах 2. Цээжээр өвдөх 3. Хоолой хорсох 4. Хоолой сэрвэлзэх, загатнах 5. Хамар загатнах 6. Хамар битүүрэх 7. Нус гоожих 8. Амьсгаадах 9. Амьсгалахад хэцүү байх 10. Нүд бүрэлзэх 11. Нүд хорсох 12. Нүд улайх 13. Нүд загатнах 14. Нулимс гоожих 15. Толгой эргэх 16. Толгой өвдөх 17. Толгой манарах, дүйнгэтэх 18. Амархан ядрах 19. Дотор муухай оргих | | Q1 |
| 2 | Агаарын бохирдол ихтэй сүүлийн жилүүдэд та болон таны гэр бүлийнхэн **эдгээр өвчнүүдээр** өвдөж байсан уу?  **(Та олон хариулт сонгож болно)** | | 1. Ханиад 2. Харшил 3. Бронхит 4. Хатгалгаа 5. Зүрхний шигдээс 6. Артерийн даралт ихсэлт 7. Чихрийн шижин 8. Цээжний өвдөлт 9. Төрөлхийн хөгжлийн гажиг 10. Зулбалт 11. Дутуу төрөлт 12. Бага жинтэй төрөлт 13. Үр хөндөлт 14. Харвалт 15. Тархины судасны өвчин 16. Мигрейн | | Q2 |
| **Агаарын бохирдлын өртөлт ба зардал** | | | | | |
| 3 | Агаарын бохирдол ихтэй үед та болон таны гэр бүлийн хэн нэгэн **өвдөөд чөлөө авч** байсан уу? | | 1. Хүүхэд өвдөөд 2. Ээж, аав өвдөөд 3. Өөрөө өвдөөд 4. Би өөрөө өвдсөн ч чөлөө авдаггүй 5. Байхгүй | | Q3 |
| 4 | Хэрэв өвдсөн бол **ямар өвчнөөр** өвдсөн бэ? | | Бичнэ үү_________________________  __________________________________ | | Q4 |
| 5 | Өнгөрсөн өвөл агаарын бохирдол ихтэй үед өвдөөд дунджаар **хэдий хугацаанд** ажилдаа ирээгүй байдаг вэ? | | 1. Цагаар__________________бичнэ үү 2. Хоногоор________________бичнэ үү 3. Ажилдаа өвдсөн ч ирдэг 4. Өвдөж байгаагүй | | Q5 |
| 6 | Өнгөрсөн өвөл агаарын бохирдол ихтэй үед өвдөөд **хэдэн удаа** ажилдаа ирээгүй байдаг вэ? | | 1. 1 удаа 2. 2 удаа 3. 3 удаа 4. 4 ба түүнээс дээш 5. Өвдөж байгаагүй | | Q6 |
| 6_1 | Та **1 өдөр** өвдөөд ажилдаа ирээгүй улмаас **хэдэн төгрөгийн** цалингаа алддаг вэ? | | Бичнэ үү ________________________төг | | Q6_1 |
| 7 | Та агаарын бохирдол ихтэй үед өвдсөний улмаас ажлын чөлөөг **хэрхэн** авдаг вэ?  **(Та олон хариулт сонгож болно)** | | 1. Цалинтай чөлөө 2. Цалингүй чөлөө 3. Ээлжийн амралтын хоногоос хасуулдаг 4. Ээлжийн амралтаа авдаг | | Q7 |
| 8 | Агаарын бохирдол ихтэй үед хамт ажилладаг хүн тань өвдөөд ажлыг нь та **орлон гүйцэтгэж** байсан уу? | | 1. Ихэнхдээ 2. Заримдаа 3. Хааяа 4. Огт үгүй | | Q8 |
| 9 | Хэрэв тийм бол тухайн хүний ажлыг орлон хийсний улмаас **стрессдэж** байсан уу? | | 1. Ихэнхдээ 2. Заримдаа 3. Хааяа 4. Огт үгүй | | Q9 |
| 10 | Агаарын бохирдол ихтэй үед ажлаас чөлөө авах талаар байгууллагадаа гэнэт мэдэгдэх үед танд **ямар санагдда**г вэ? | | 1. Айж, эмээдэг 2. Санаа зовдог 3. Тайван, санаа зовох зүйлгүй байдаг 4. Бусад_______________________ | | Q10 |
| 11 | Та өвдөхөд байгууллагаас **уян хатан зохицуулалт** (гэрээсээ ажлаа хийх, ажлыг орлон гүйцэтгүүлэх г.м) хийдэг үү? | | 1. Ихэнхдээ 2. Заримдаа 3. Хааяа 4. Огт үгүй | | Q11 |
| 12 | Агаарын бохирдол их үед танай гэр бүлээс **ХЭН** хамгийн өвчлөмтгий вэ?  /Та зөвхөн нэг хариулт сонгоно уу/ | | 1. Би өөрөө 2. Жирэмсэн эмэгтэй 3. Эхнэр, нөхөр 4. Хүүхдүүд 5. Эцэг,эх 6. Эмээ, өвөө | | Q12 |
| **Дээрх 12-р асуултад сонгосон** хүнд гардаг зардлыг бичнэ үү.  Өнгөрсөн өвлийн талаар бодож бичнэ. | | | Хариулт | |  |
|  |  |  | Зардлын давтамж  /удаа/ | Дундаж зардал /төгрөгөөр/ |  |
| 13 | Оношилгоо шинжилгээтэй холбоотой зардал | | __ __ удаа | ___________төг | Q13A |
|  | Эм худалдан авсан зардал | | __ __ удаа | ___________төг | Q13B |
|  | Эмнэлэгт хэвтэн эмчлүүлсэн зардал | | __ __ удаа | ___________төг | Q13C |
|  | Тээвэр зардал | | __ __ удаа | ___________төг | Q13D |
| **Хүн ам зүйн мэдээллийн хэсэг** | | | | | |
| 14 | | Таны нас | __ __ | | Q14 |
| 15 | | Таны хүйс | 1. Эрэгтэй 2. Эмэгтэй | | Q15 |
| 16 | | Таны боловсрол | 1. Дээд 2. Дунд 3. Бага | | Q16 |
| 16.1 | | Сурсан жил | ________жил | | Q16_1 |
| 17 | | Таны албан тушаал | __________________________________ | | Q17 |
| 17_1 | | Та сарын цалингийн дундаж хэмжээгээ сонгоно уу? | 1. <399.000 төг 2. 400.000- 699.000 төг 3. 700.000- 999. 000 төг 4. 1.000.000 – 1.299.000 төг 5. 1.300.000 – 1.599.000 төг 6. 1.600.000- 1.899.000 төг 7. 1.900.000 - 2.199.000 төг 8. 2.200.000 дээш төг | | Q17A |
| 18 | | Таны тухайн байгууллагад нийт ажилласан жил | Q18.1 __ __ жил, Q18.2 __ __ сар | | Q18 |
| 19 | | Таны гэрийн хаяг  Гэрийн хаягийн мэдээллээр хувь хүний агаарын бохирдлын өртөлтийг тооцоолох боломжтой. | ______________________дүүрэг ___________хороолол_______ хороо_____байр/ хашаа_______ гудамж__________________тоот | | Q19 |
| 20 | | Хүүхэдтэй эсэх | 1. Тийм   2. Үгүй | | Q20 |
| 21 | | Тийм бол хүүхдийн тоо | ___ ___ | | Q21 |
| 22 | | Та өдөрт хэдэн цаг ажилладаг вэ? | __ __ | | Q22 |
| 23 | | Таны гэрээс ажил хүртэлх зай | ___ ___ км __ __ Автобусны буудлын тоо | | Q23 |
| 24 | | Та өвлийн улиралд **шүүлтүүртэй маск** зүүдэг үү? | 1. Ихэнхдээ 2. Заримдаа 3. Санахаараа зүүдэг 4. Ерөөсөө зүүдэггүй | | Q24 |
| 25 | | Та өвлийн улиралд **гэртээ агаар цэвэршүүлэгч** хэрэглэдэг үү? | 1. Ихэнхдээ 2. Заримдаа 3. Хааяа 4. Огт үгүй | | Q25 |
| 26 | | Агаарын бохирдол ихтэй үед **байгууллагын зүгээс** ямар арга хэмжээ авдаг талаар сонгоно уу. | 1. Шүүлтүүртэй маск тараадаг 2. Өрөө бүрд агаар цэвэршүүлэгч тавьж өгдөг 3. Аарц, чацаргана өгдөг 4. Арга хэмжээ авдаггүй 5. Бусад____________________ | | Q26 |
| 27 | | Таны амьдардаг орон сууц? | 1. Гэр 2. Хашаа, байшин 3. Орон сууц 4. Нийтийн сууц 5. Бусад ___________________ | | Q27 |
| 27_1 | | Та өнгөрсөн өвөл **ямар төрлийн халаалт** хэрэглэдэг байсан бэ? | 1. Нийтийн халаалт 2. Сайжруулсан түлш 3. Түүхий нүүрс 4. Цахилгаан халаагуур 5. Мод 6. Бусад ___________________ | | Q27_1 |
| 28 | | Та ажилдаа яаж ирдэг вэ? | 1. Алхдаг 2. Унадаг дугуйгаар 3. Машинаар 4. Нийтийн автобусаар 5. Байгууллагын унаагаар 6. Бусад | | Q28 |
| 29 | | Та тамхи татдаг уу? | 1. Тийм 2. Үгүй | | Q29 |
| 29_1 | | Тийм бол тамхийг **хэдэн жил, өдөрт хэдэн ширхгийг** татаж байна вэ? | 1. Жил __ __ 2. Өдөрт татдаг тоо ___ ___ | | Q29_1 |
| 29_2 | | Үгүй бол та гэртээ болон ажлын газраа **дам тамхидалтад** өртдөг үү? | 1. Ихэнхдээ 2. Заримдаа 3. Огт үгүй | | Q29_2 |
| 30 | | Таны биеийн жин, өндөр?  /Биеийн жингийн индексийг тооцоолоход ашиглана/ | 1. Өндөр__ __ __ см 2. Жин___ ____ кг | | Q30 |
