# Supplementary material for: Integrating quantitative and qualitative approaches to assess wintertime illness-related absenteeism and its direct and indirect costs among the private sector in Ulaanbaatar
Source: PLoS One. 2022 Feb 3;17(2):e0263220. doi: 10.1371/journal.pone.0263220 (PMC8812901; doi:10.1371/journal.pone.0263220)
Supplement: S2 File — (DOCX) [file pone.0263220.s002.docx]

**Individual interview moderator’s guide questions**

**Section 1: Sickness related absenteeism questions**

1.1 What do you think about employee and employer absenteeism status?

1.2 Please speak about absenteeism due to air pollution-related diseases in your company?

- Please address this in detail according to differences in sex, workload and having children.
- How often company employees absent from the work? Did you observe any frequent cases with similar reason where certain employees often request the work leave?
- What kind of flexible working arrangements were offered by your company? Tell me about this

**Section 2: Company direct and indirect costs due to air pollution-related absenteeism**

2.1 What kind of expenses concur in your company when employees feel sick and request unplanned leave? Are these expenses going to increase?

2.2 Does the company have strategy to reduce the costs due to absenteeism?

2.3 Please explain in detail about indirect costs due to absenteeism in winter months

- The cost of outsourcing to cover the labor shortage
- Overtime for replacement of absent worker
- Training expenses for temporary/replacement workers
- Loss of production and quality of product/service due to absenteeism
- Reduced quality performance by under-trained replacement workers
- Employees stress due to duty sharing
- Management cost related to dealing with absenteeism
- How the company calculates the productivity loss?

**Section 3: The company air pollution coping strategies**

| **The company air pollution coping strategies** | **Yes** | **No** |
| --- | --- | --- |
| Whether to distribute air pollution masks to their employee |  |  |
| Whether to allocate air pollution masks to their employees’ children |  |  |
| Whether to locate air purifier in each office room | If yes, total __ |  |
| Whether to vaccinate employees against influenza and influenza-like illnesses |  |  |
| Whether to organize preventive examination for employees | ___ in year ___ times |  |
| Whether the company has its doctor |  |  |
| Whether the company has its hygienist |  |  |
| Whether distribute dried milk and sea buckthorn to their employees |  |  |
| Whether the company organize the training on air pollution and health prevention |  |  |
| Whether the company owners wear air pollution mask themselves |  |  |

**Ганцаарчилсан ярилцлагын чиглүүлэх асуултууд**

**1. Өвчлөлөөс шалтгаалсан ажилдаа ирээгүй байдлыг тодруулах хэсэг**

1.1 Байгууллагын ажилтан, ажиллагсдын ажилдаа ирээгүй байдлын талаар та юу гэж бодож байна вэ?

1.2 Та агаарын бохирдлоос үүдэлтэй өвчлөлтэй холбоотой ажилдаа ирээгүй байдлын талаар ярина уу?

- Хүйсээр, ажлын ачаалал болон хүүхэдтэй зэргээс хамааран ямар байдаг талаар дэлгэрүүлэн асууна.
- Компанийн ажилчдын ажил таслалтын давтамж нь ямар байдаг вэ? Та тооцоолол хийх явцдаа нэг хүн нэг шалтгааны улмаас олон давтамжаар чөлөө авч байсан тохиолдлыг ажиглаж байсан уу?
- Танай компани ажилтнууддаа зориулан ажиллах уян хатан цагийн зохицуулалт хийдэг үү? /Жишээ нь танай ажилтан өөрөө эсвэл ойр дотных нь хүн өвдсөн тохиолдолд тухайн ажилтан гэрээсээ ажиллах эсвэл ажлын бус цагаар ажлаа хийх, ажлыг хэн нэгэн орлон гүйцэтгэх боломжтой эсэх талаар/

**2. Компанийн ажилтан ажилдаа ирээгүй байдлаас шалтгаалсан байгууллагад учрах зардлыг тодруулах хэсэг**

2.1 Танай компанийн ажилтан өвдсөн тохиолдолд чөлөө авах үед компанийн талаас ямар зардлууд гардаг талаар ярина уу. Энэ зардал цаашдаа өсөх хандлагатай байна уу?

2.2 Ажил таслалттай холбоотойгоор бий болж байгаа зардлыг буруулах менежмент байдаг уу?

2.3 Байгууллагад учрах шууд бус зардлыг тодруулах хэсэг

- Ажиллах хүчний дутагдалтай байдлыг нөхөхтэй холбоотой зардалууд
- Байхгүй ажилтныг орлож илүү цагаар ажилласнаас үүдсэн зардал
- Байхгүй ажилтныг орлогч ажилтныг сургах, дадлагажуулахтай холбоотой зардал
- Ажил таслалтаас үүдэн бүтээгдэхүүн, үйлдвэрлэл болон үйлчилгээнд учирсан хохирол
- Чөлөөтэй ажилтныг орлох ажилтныг олох, сургах гэх мэт үйл ажиллагаатай холбоотой удирдлагын зардал
- Орлогч ажилтанаас үүдэн гүйцэтгэлийн чанар буурсан байдал
- Чөлөөтэй ажилтныг орлох байдлаас бусад ажилтнуудад үүссэн стресс
- Танай компани бүтээмж алдагдлыг хэр тооцдог вэ?

**3. Танай байгууллага ажилчдаа агаарын бохирдлоос сэргийлэхийн тулд ямар арга хэмжээ авдаг вэ?**

| **Байгууллага агаарын бохирдлоос сэргийлэхийн тулд ажилчдадаа зориулж авдаг арга хэмжээнүүд** | **Тийм** | **Үгүй** |
| --- | --- | --- |
| Шүүлтүүртэй маск тараадаг эсэх |  |  |
| Ажилчдын хүүхдүүдэд шүүлтүүртэй маск тараадаг эсэх |  |  |
| Байгууллагын өрөө бүрт агаар цэвэршүүлэгч байрлуулсан эсэх | Тийм бол нийт тоо __ |  |
| Ажилчдыг томуу болон томуу төст өвчний вакцинд хамруулдаг эсэх |  |  |
| Ажилчдыг эрүүл мэндийн урьдчилан сэргийлэх үзлэгт хамруулдаг эсэх | ___ жилд ___ удаа |  |
| Компанид өөрийн эмч байгаа эсэх |  |  |
| Компанид эрүүл ахуйч байгаа эсэх |  |  |
| Ажилчдын эрүүл мэндийг хамгаалах зорилгоор аарц, чацаргана өгдөг эсэх |  |  |
| Ажилчдаа агаарын бохирдолтой холбоотой өвчлөлөөс сэргийлэх зорилгоор сургалт зохион байгуулдаг эсэх |  |  |
| Компанийн удирдлагууд өөрөө шүүлтүүртэй маск зүүдэг эсэх |  |  |
