# Supplementary material for: Integrating quantitative and qualitative approaches to assess wintertime illness-related absenteeism and its direct and indirect costs among the private sector in Ulaanbaatar
Source: PLoS One. 2022 Feb 3;17(2):e0263220. doi: 10.1371/journal.pone.0263220 (PMC8812901; doi:10.1371/journal.pone.0263220)
Supplement: S3 File — (DOCX) [file pone.0263220.s003.docx]

# Focus group questions to moderators

**Section 1: To clarify the air pollution related diseases**

1.1 What do you think about air pollution in Ulaanbaatar?

1.2 Please address about diseases related to air pollution.

1.3 How many times and how long have you been sick during winter highest air pollution period?

**Section 2: Sick leave during high polluted winter months**

2.1 Have you ever been absent during the high air pollution period? If yes, please address this in detail. What are the main causes when you don’t come to your office due to sickness?

2.2 Have you ever been absent from the work due to air pollution-related diseases?

2.3 Who is the sickest person in your household during winter's high air pollution periods?

2.4 Does your company offer flexible working arrangement when you or your family member request sudden leave due to illness? For instance, work from home, replacing your duty by someone in office etc.

2.5 How do you feel when you request sudden leave from your job due to illness during high air pollution in winter? Moreover, please say about possible alternatives to request short-time leave from the job.

**Section 3: Costs related to absenteeism due to illness**

3.1 Please say about the costs due to sickness on high air pollution days. /An average expenses related to purchasing medicine, diagnosis and doctor visits, transportation and salary deduction/

3.2 How much expenses of treatment and diagnosis do you spend when you and one of your family members get sick during the high air pollution period?

3.3 How much costs when you are sick to buy medicines during winter time?

3.4 Do you receive premium from health and social insurance?

**Section 4: Individual air pollution prevention behavior**

| **Individual air pollution prevention behavior** | **Yes**  **/Number of participants/** | **No**  **/Number of participants/** |
| --- | --- | --- |
| Do you wear air pollution mask during high air pollution period? |  |  |
| Do you purchase air pollution mask to your children? |  |  |
| Do you use air purifier at home? |  |  |
| Have you got vaccinated against influenza and influenza-like illness? |  |  |
| Do you participate in preventive health examinations? | ___year ___ times |  |
| Do you pass sea buckthorn or lingonberry juices, and fermented hot, dry milk and vitamins to your children? |  |  |

**Section 5: End of the questions**

5.1 If you are willing to add any information related to that topic, please conclude as what you think

# Модераторын асуултууд

**1. Агаарын бохирдолтой холбоотой өвчлөлийн талаар тодруулах хэсэг**

1.1 Та агаарын бохирдлын талаар ярина уу.

1.2 Агаарын бохирдолтой холбоотой өвчлөлийн талаар ярина уу.

1.3 Та өвлийн улиралд агаарын бохирдол өндөр үед хэр давтамжтай, хэр удаан хугацаагаар өвддөг вэ?

**2. Агаарын бохирдол ихтэй үед өвдөөд ажилдаа ирээгүй талаар тодруулах хэсэг**

2.1 Агаарын бохирдол ихтэй үед ажилдаа ирээгүй талаар ярина уу, гол шалтгааныг та юу гэж бодож байна вэ?

2.2 Агаарын бохирдлоос үүдэлтэй өвчний улмаас ажилдаа ирээгүй тохиолдол байгаа юу?

2.3 Агаарын бохирдол ихтэй үед та болон таны гэр бүлийн хэн их өвдөж харах, асрах шаардлага гардаг вэ?

2.4 Агаарын бохидол ихтэй үед таны гэр бүлийн хэн нэгэн өвдөж гэнэтийн чөлөө авах үед байгууллагын зүгээс уян хатан зохицуулга хийдэг үү. Тухайлбал, гэрээсээ ажиллах, хэн нэгэн таны оронд нөхөн ажиллах гэх мэт

2.5 Агаарын бохирдол өндөр үед гэнэт өвдсөний улмаас байгууллагаас чөлөө авахад танд хүндрэлтэй санагддаг уу. Чөлөө богино хугацаанд авах боломжийн талаар дэлгэрэнгүй ярина уу.

**3. Өвдөөд ажилдаа ирээгүй үед гарах зардлуудын талаар**

3.1 Агаарын бохирдол ихтэй үед өвдөснөөс болж гардаг зардлуудын талаар нээлттэй асууна.

Цалин, мөнгө, эмчилгээ, эм, унаа, хоолны мөнгөний талаар дэлгэрүүлэн асууна.

3.2 Та өвлийн улиралд өвдсөнөөс болж эмчилгээ, оношилгоотой холбоотой хэр их зардал ойролцоогоор гаргадаг вэ?

3.3 Та өвлийн улиралд өвдсөнөөс болж эм худалдан авахтай холбоотой хэр их зардал ойролцоогоор гаргадаг вэ?

Танд болон танай гэр бүлд тус, тусад нь ялгаж асууна.

3.4 Эрүүл мэндийн даатгал болон нийгмийн даатгалаас оношилгоо, хөнгөлөлт авдаг эсэх

**4. Агаарын бохирдлын өртөлтөөс өөрийгөө болон гэр бүлээ хамгаалахын тулд та ямар арга хэмжээ авдаг вэ?**

| **Агаарын бохирдлын өртөлтөөс сэргийлэхийн тулд авдаг арга хэмжээнүүд** | **Тийм**  **/хүний тоо/** | **Үгүй**  **/хүний тоо/** |
| --- | --- | --- |
| Өвлийн улиралд шүүлтүүртэй маск зүүдэг эсэх |  |  |
| Хүүхдүүддээ зориулж агаарын бохирдлын маск худалдан авдаг эсэх |  |  |
| Гэртээ агаар цэвэршүүлэгч хэрэглэдэг эсэх |  |  |
| Томуу болон томуу төст өвчний вакцинд хамрагдсан эсэх |  |  |
| Урьдчилан сэргийлэх үзлэгт хамрагддаг эсэх | ___жилд ___ удаа |  |
| Өвлийн улиралд хүүхдүүддээ аарц, чацаргана, амин дэм өгдөг эсэх |  |  |

**5. Бүлгийн ярилцлагыг хаах асуултууд**

5.1 Бид та бүхэнтэй агаарын бохирдлоос үүдэлтэй ажилдаа ирээгүй байдал, түүний зардлуудын талаар ярилцлаа. Та бүхэн бидний ярилцсан сэдвүүдээс дүгнэж хэлнэ үү?
